# Effect of Sucralose Consumption on Gut Microbiota: A Systematic Review and Meta-Analysis of Randomized Controlled Trials

**DOI:** 10.3390/ijms27188377

**Published:** 2026-09-20

**Authors:** Dwi Tari Wulandari, Nuri Andarwulan, Saraswati, Eny Palupi, Dominika Średnicka-Tober

**Affiliations:** 1 Division of Food Science and Technology, Faculty of Engineering and Technology, IPB University, IPB Dramaga Campus, Bogor 16680, Indonesia; dwitariwulandari@gmail.com (D.T.W.); saraswati-sa@apps.ipb.ac.id (S.); 2 South-East Asia Food & Agricultural Science and Technology (SEAFAST) Center, IPB University, IPB Dramaga Campus, Bogor 16680, Indonesia; 3 Department of Community Nutrition, Faculty of Medicine and Nutrition, IPB University, IPB Dramaga Campus, Bogor 16680, Indonesia; enypalupi@apps.ipb.ac.id; 4 Department of Functional and Organic Food, Institute of Human Nutrition Sciences, Warsaw University of Life Sciences, Nowoursynowska 159c, 02-776 Warsaw, Poland

**Keywords:** gut microbiota, meta-analysis, sucralose, randomized controlled trial

## Abstract

Sucralose is a widely consumed non-nutritive sweetener, with approximately 85% reaching the colon unmetabolized and directly interacting with the gut microbiota. Despite regulatory approval, its impact on microbial ecology and metabolic outcomes remains inconclusive. Previous meta-analyses included only two human studies and did not integrate microbiota, short-chain fatty acids, and metabolic parameters. This systematic review and meta-analysis evaluated the effects of sucralose on gut microbiota composition, alpha diversity, short-chain fatty acid production, and metabolic parameters in healthy adults based on randomized controlled trial evidence. A systematic search of PubMed, Scopus, and Google Scholar was conducted in May 2026 following PRISMA guidelines. Risk of bias was assessed using Cochrane RoB 2.0, and meta-analysis employed a REML random-effects model. Ten randomized controlled trials involving 8–61 participants per study (total across intervention and control groups) met the inclusion criteria; seven provided data for meta-analysis. Sucralose did not significantly alter alpha diversity (Shannon index: SMD = −0.00; 95% CI −1.16 to 1.15; *p* = 0.99; I^2^ = 25.67%), fasting blood glucose (SMD = −0.04; 95% CI = −0.63 to 0.56; *p* = 0.88; I^2^ = 69.61%), and HOMA-IR (SMD = −0.13; 95% CI = −0.36 to 0.10; *p* = 0.212; I^2^ = 0.00%). Butyrate showed a numerical decrease, although the pooled effect was not statistically significant and substantial heterogeneity was observed. Overall, the available evidence from short-term randomized controlled trials did not demonstrate statistically significant effects of sucralose consumption on the evaluated microbiota and metabolic outcomes. Standardized, longer-duration randomized controlled trials with inert comparators are needed.

## 1. Introduction

Artificial sweeteners have become increasingly popular as food additives in the modern food and beverage industry, serving as sugar substitutes in low-calorie and diet products. This trend is reflected in the continuous growth of the global artificial sweetener market between 2021 and 2025, with sales projected to increase at a compound annual growth rate (CAGR) of 7.25% from 2025 to 2033 [1]. Among the available artificial sweeteners, sucralose is one of the most widely used due to its intense sweetness, which is approximately 600 times greater than that of sucrose, while providing virtually no calories [2]. Consequently, sucralose has been extensively incorporated into a wide range of food and beverage products, including diet soft drinks, bakery products, packaged foods, and nutritional supplements. The safety of sucralose has been evaluated and approved by several regulatory authorities, including the Food and Drug Administration (FDA) and the European Food Safety Authority (EFSA). These agencies have established acceptable daily intake (ADI) values of 5 mg/kg body weight/day and 15 mg/kg body weight/day, respectively [3]. Despite its widespread use and regulatory approval, the long-term health effects of sucralose consumption, particularly its impact on the gut microbiota, remain a subject of ongoing scientific investigation and debate.

The gut microbiota comprises trillions of microorganisms that play essential roles in maintaining human health, including energy metabolism, immune function, and the regulation of hormonal and inflammatory responses [4]. Alterations in gut microbial composition have been associated with the development of several chronic diseases, including obesity, type 2 diabetes, inflammatory bowel disease, and cardiovascular disease [5]. Among the predominant bacterial phyla in the human gut, Actinobacteria, Bacteroidetes, and Firmicutes have been the most extensively investigated because of their pivotal roles in maintaining intestinal homeostasis and metabolic health [6]. Ahmad et al. [6] reported a significant alteration (*p* < 0.05) in the composition of two major gut bacterial phyla, Bacteroidetes (genus *Bacteroides*) and Firmicutes (order *Clostridiales*), following a 14-day intervention with sucralose administered as a beverage at a dose equivalent to approximately 20% of the acceptable daily intake (ADI; ±136 mg/day). Similarly, evidence from experimental studies and randomized controlled trials (RCTs) suggests that artificial sweeteners, including sucralose, may disrupt gut microbial homeostasis by reducing the abundance of beneficial bacteria while promoting the proliferation of specific opportunistic microorganisms. These alterations may subsequently impair metabolic function and immune regulation, thereby potentially increasing the risk of adverse health outcomes [7].

Several studies have suggested that sucralose consumption may increase intestinal permeability, commonly referred to as “leaky gut syndrome,” thereby facilitating the translocation of microbial-derived compounds into the systemic circulation. This process may trigger systemic inflammatory responses and subsequently increase the risk of various chronic diseases [5]. Furthermore, sucralose-induced alterations in the gut microbiota have been associated with impaired insulin sensitivity, potentially contributing to the development of type 2 diabetes [4]. However, these findings remain inconsistent. For example, Thomson et al. [8] reported that high-dose sucralose consumption for seven consecutive days did not result in significant alterations in gut microbiota composition, highlighting the variability in the reported effects of sucralose across clinical studies.

Given the increasing consumption of food products containing sucralose, a comprehensive evaluation of its effects on the gut microbiota is necessary. Meta-analysis provides a robust framework for synthesizing available evidence by integrating data from multiple randomized controlled trials (RCTs), thereby enhancing the precision and reliability of the pooled estimates. A previous systematic review and meta-analysis conducted by Chen et al. [9] evaluated the effects of sucralose on the gut microbiota in both humans and rodents to compare species-specific responses. Their findings demonstrated alterations in the abundance of two major bacterial phyla and a reduction in obesity-related outcomes in humans. However, the human meta-analysis was based on only two eligible studies, which limits the precision of its estimates and the certainty of the available evidence. Since the publication of the previous review, several additional randomized controlled trials have investigated the effects of sucralose in humans. Incorporating these newly published studies is expected to strengthen the evidence base and provide a more reliable assessment of the impact of sucralose consumption on gut microbiota composition and metabolic outcomes. Therefore, the present systematic review and meta-analysis were conducted to provide an updated and comprehensive synthesis of the available evidence by evaluating the effects of sucralose consumption on gut microbiota composition and metabolic responses in healthy adults. We hypothesized that sucralose consumption may alter gut microbiota composition and metabolic responses in healthy adults.

## 2. Materials and Methods

### 2.1. Study Period and Data Sources

This study was conducted from June 2025 to May 2026 at the Division of Food Science and Technology, Faculty of Engineering and Technology, IPB University. Secondary data were collected from published studies retrieved through scientific literature databases, including Scopus, PubMed, and Google Scholar. Publish or Perish was used as a literature search application.

### 2.2. Study Design and Protocol

This study was conducted as a systematic review and meta-analysis following the Preferred Reporting Items for Systematic Reviews and Meta-Analyses (PRISMA 2020) guidelines [10]. The PRISMA 2020 Checklist is provided in the Supplementary Materials (Table S1). The review protocol was prospectively registered in the International Prospective Register of Systematic Reviews (PROSPERO) under registration number CRD420261422049. A comprehensive literature search was performed using PubMed, Scopus, and Google Scholar to identify studies published within the last ten years. A literature search was performed using combinations of keywords related to sucralose and gut microbiota as follows: (“sucralose” OR “Splenda” OR “non-nutritive sweetener”) AND (“gut microbiota” OR “intestinal microbiome” OR “gut flora” OR “fecal microbiota”) AND (“clinical trial”). A complete search strategy is presented in Table S2 (Supplementary Materials).

### 2.3. Inclusion and Exclusion Criteria

Study eligibility was determined according to the PICO framework. The population included healthy adults aged 18 years or older. The intervention consisted of oral sucralose consumption, while the comparison group received a placebo or control treatment. The primary outcome was changes in gut microbiota composition, particularly the Firmicutes-to-Bacteroidetes (F/B) ratio and related microbiota-derived metabolites, including short-chain fatty acids (SCFAs). Studies were excluded if they evaluated mixtures of sweeteners without reporting separate data for sucralose; employed non-randomized controlled trial (non-RCT) designs; included participants with underlying diseases; were exclusively in vitro experiments; or were conducted using animal models.

### 2.4. Study Selection and Data Extraction

Study selection was independently performed by two reviewers through title and abstract screening, followed by full-text eligibility assessment with RayyanAI (Rayyan Systems Inc., Cambridge, MA, USA). The extracted data included study characteristics, intervention details (sucralose dose, intervention duration, and comparator/placebo type), and outcome variables. For studies reporting the mean difference and standard deviation (SD), effect sizes were calculated using Hedges’ g. A random-effects model was applied using the restricted maximum likelihood (REML) method to estimate between-study variance (τ^2^), with the Hartung–Knapp adjustment applied to calculate the confidence intervals and statistical significance of the pooled effects. The pooled results were presented as forest plots, and between-study heterogeneity was quantified using the I^2^ statistic. Publication bias was not formally assessed because fewer than 10 studies were available for each meta-analyzed outcome. Quantitative analyses were performed using Microsoft Excel, JASP version 0.96, and R version 4.5.3 with the “metafor” package version 5.0-1. Statistical power was assessed using a post hoc approach based on the framework described by Hedges and Pigott for power analysis in meta-analysis. Power was calculated using the pooled Hedges’ g and its corresponding standard error, assuming a two-sided significance level of 0.05. The analysis was used to assess the ability of the available evidence to detect effects of the observed magnitude.

### 2.5. Risk of Bias Assessment

The risk of bias was assessed using the Cochrane Risk of Bias 2.0 (RoB 2.0) tool across five domains: D1 (bias arising from the randomization process), D2 (bias due to deviations from intended interventions), D3 (bias due to missing outcome data), D4 (bias in the measurement of the outcome), and D5 (bias in the selection of the reported result). The risk-of-bias assessment is illustrated in Table S3. 

The certainty of evidence was assessed using the GRADE approach (Table S4).

## 3. Results

### 3.1. Characteristics of the Studies Included in the Systematic Review and Meta-Analysis

**Figure 1:**
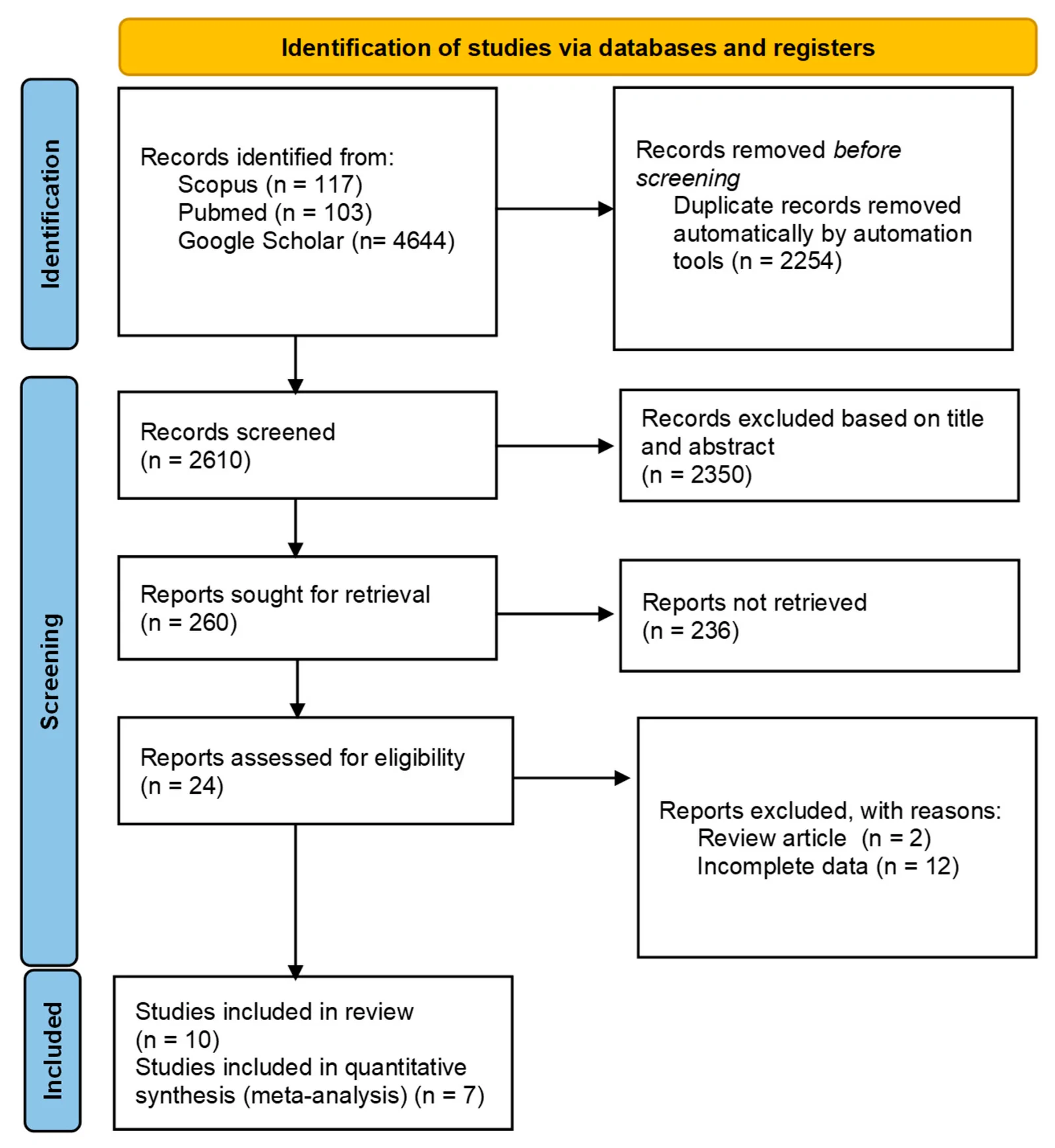
PRISMA 2020 flow diagram of the study selection process.

The literature search and study selection process conducted in accordance with the PRISMA 2020 guidelines identified 10 eligible studies for data extraction. The complete study selection process is illustrated in Figure 1.

A total of 4864 records were retrieved from three electronic databases, comprising Scopus (n = 117), PubMed (n = 103), and Google Scholar (n = 4644). Duplicate records (n = 2254) were automatically identified and removed using Rayyan AI. Following duplicate removal, 2610 studies underwent title and abstract screening based on the predefined inclusion criteria. Of these, 24 articles were considered eligible for full-text assessment. After evaluating the full-text articles for data completeness and compliance with the inclusion criteria, 10 studies were included in the systematic review. Among these, seven studies provided sufficient quantitative data for meta-analysis of alpha diversity, short-chain fatty acids (SCFAs), fasting blood glucose, and HOMA-IR.

Table 1 presents the characteristics of the sucralose consumption studies included in the systematic review. Approximately 70% of the included studies were conducted in North America, while the remaining studies originated from South America, Southeast Asia, and West Asia. This geographical distribution likely reflects the higher prevalence of artificial sweetener consumption, particularly in the form of sugar-sweetened and diet soft drinks, within North American populations [11].

The included studies primarily enrolled healthy adults with a normal body mass index (BMI), ranging in age from 18 to 70 years. Sample sizes varied considerably across studies, as did the intervention protocols and comparator formulations. The diversity of comparators and intervention types represented one of the major methodological challenges identified in this systematic review. In particular, substantial heterogeneity was observed in the comparator used among studies, which may have contributed to variability in the reported outcomes. The comparators used across the included studies varied in composition and were descriptively classified into sugar-free and sugar-containing categories.

### 3.2. Risk of Bias

**Figure 2:**
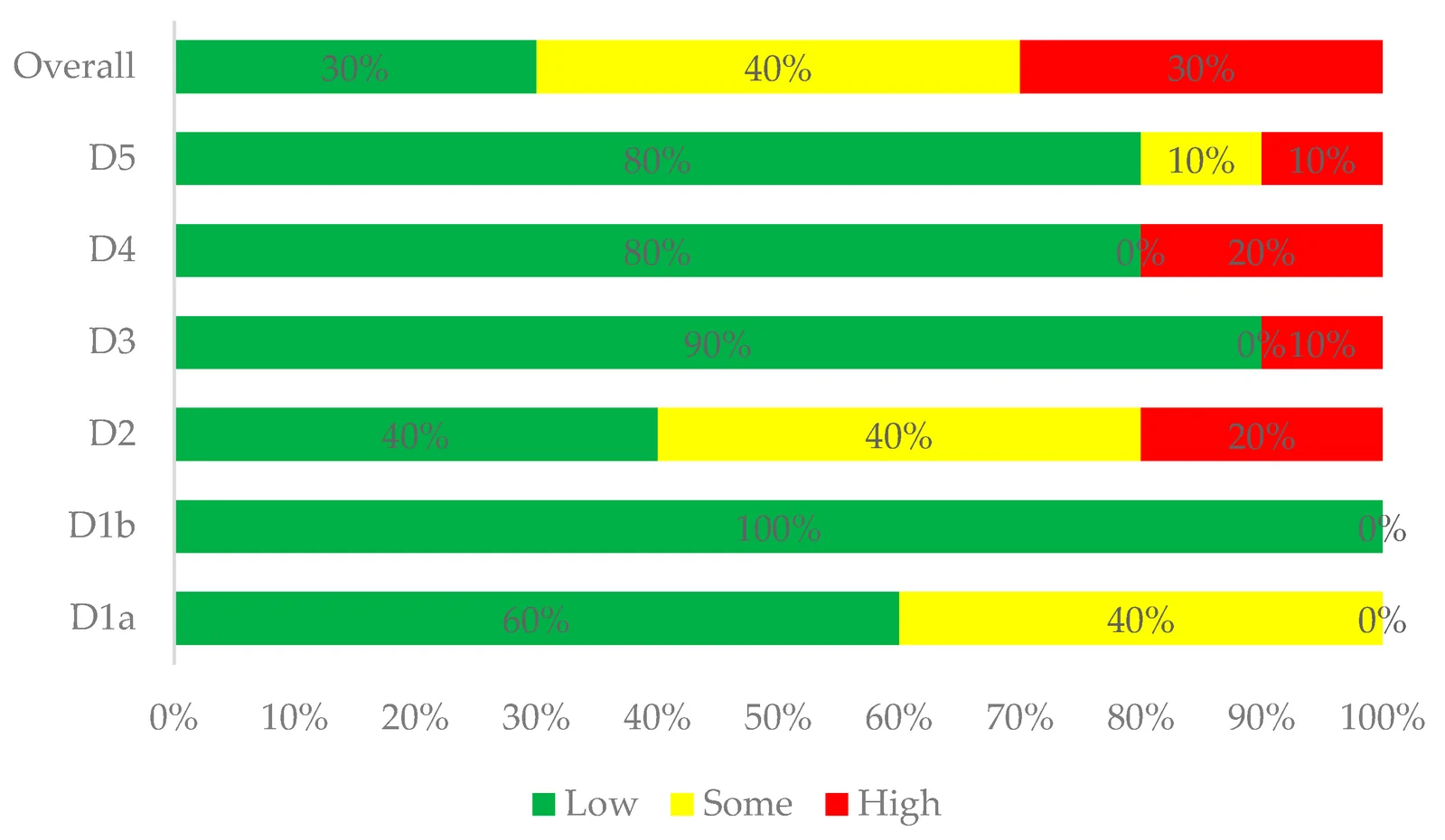
Summary of risk-of-bias assessment across the included randomized controlled trials using the Cochrane RoB 2 tool. D1a = bias arising from the randomization process (random sequence generation); D1b = bias arising from the randomization process (allocation concealment); D2 = bias due to deviations from intended interventions; D3 = bias due to missing outcome data; D4 = bias in the measurement of the outcome; D5 = bias in the selection of the result.

The risk of bias was evaluated using the Cochrane Risk of Bias (RoB 2.0) tool across five domains (D1–D5). The overall assessment showed that 30% of the included studies were classified as having a high risk of bias, 40% raised some concerns, and 30% were considered to have a low risk of bias (Figure 2). The primary source of high risk of bias was identified in Domain 2 (deviations from intended interventions), mainly due to differences in placebo formulations and blinding procedures across studies. Randomized controlled trials generally assume that the placebo is biologically inert and does not influence the measured outcomes. However, the present review demonstrated that this assumption was not consistently met, as several placebo formulations may have exerted biological effects on the gut microbiota, potentially introducing bias into the estimated effects of sucralose.

### 3.3. Effects of Sucralose Consumption on Gut Microbiota and Short-Chain Fatty Acids

**Figure 3:**
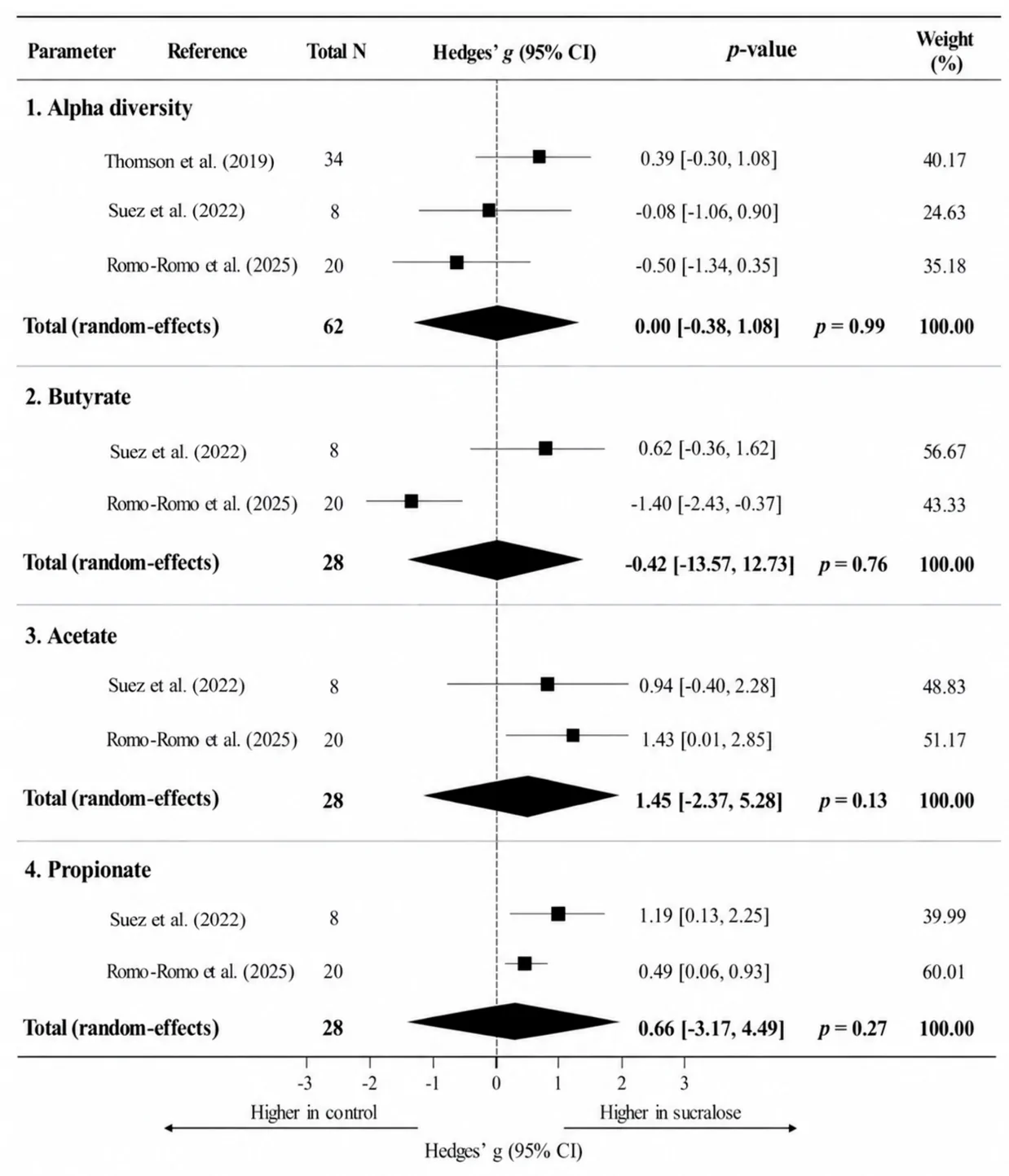
Forest plots of the effects of sucralose consumption on gut microbiota composition and short-chain fatty acids (SCFAs). Squares represent individual study effect sizes, horizontal lines indicate 95% CIs, and diamonds represent pooled effect sizes. No statistically significant differences were observed for any of the analyzed outcomes (*p* > 0.05) [8,14,16].

Changes in gut microbiota were evaluated using alpha diversity and short-chain fatty acid (SCFA) concentrations. The pooled meta-analysis results are presented as forest plots in Figure 3. Alpha diversity, which reflects the richness and evenness of microbial species within a community, was assessed using the Shannon diversity index. Meta-analysis of the three studies reporting this outcome indicated that sucralose consumption did not significantly affect alpha diversity compared with the control group (SMD = −0.00; 95% CI: −1.16 to 1.15; *p* = 0.99). Between-study heterogeneity was low (I^2^ = 25.67%; Q = 2.49; *p* = 0.29).

Similarly, analysis of butyrate, one of the principal SCFAs produced by gut microbiota, revealed no statistically significant difference between the sucralose and control groups, although a decreasing trend was observed (SMD = −0.42; 95% CI: −13.57 to 12.73; *p* = 0.76; I^2^ = 88.10%; *p* = 0.004). The substantial heterogeneity suggests considerable variability among studies in terms of intervention protocols, participant characteristics, and analytical methods. Acetate (SMD = 1.45; 95% CI: −2.37 to 5.28; *p* = 0.13; I^2^ = 0.00%; *p* = 0.50) and propionate (SMD = 0.66; 95% CI: −3.17 to 4.49; *p* = 0.27; I^2^ = 31.49%; *p* = 0.22) also showed no statistically significant effects. Nevertheless, the observed downward trend in butyrate may be biologically relevant and warrants further investigation despite the lack of statistical significance.

The observed reduction in butyrate production may be associated with alterations in gut microbial composition, particularly changes in microbial composition, including shifts in the *Firmicutes*-to-*Bacteroidetes* (F/B) ratio. Variations in the relative abundance of the phyla *Firmicutes* and *Bacteroidetes*, as presented in Table 2, differed across the included studies and were interpreted based on the direction and magnitude of changes in both phyla. Notably, Ahmad et al. [6] reported an increase in the F/B ratio, in contrast to the findings of the other three studies, which demonstrated either a decrease or no substantial change in this ratio.

### 3.4. Effects of Sucralose Consumption on Metabolic Response

**Figure 4:**
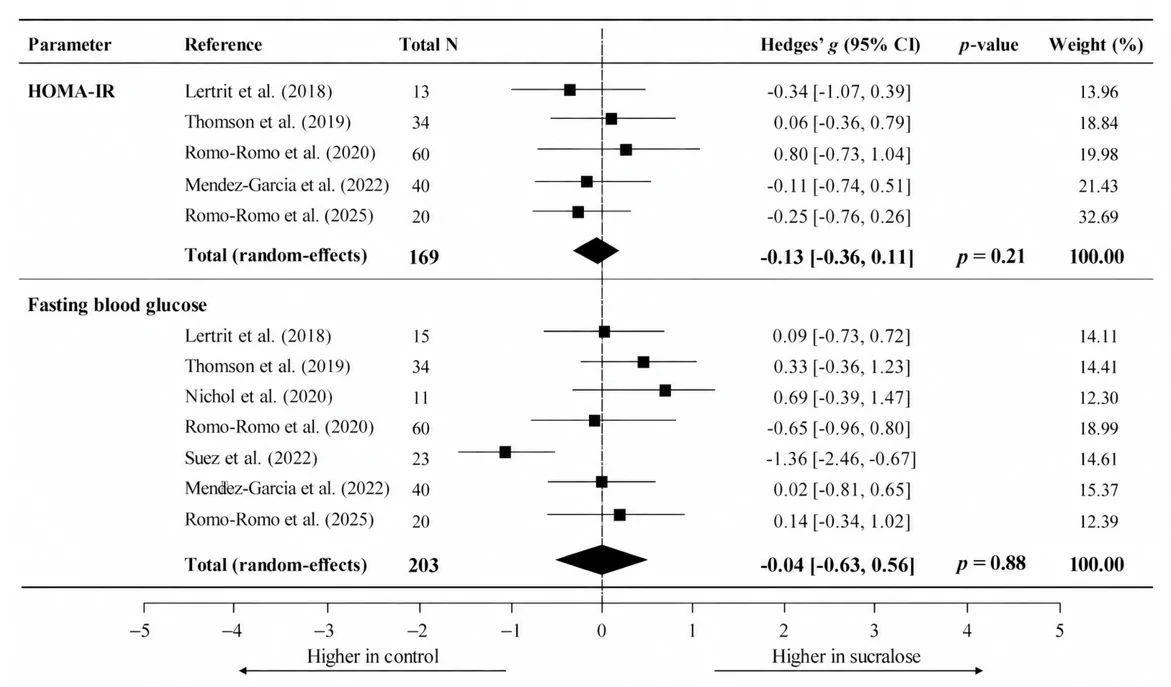
Forest plots showing the effects of sucralose consumption on fasting blood glucose and HOMA-IR. Squares represent individual study effect sizes, horizontal lines indicate 95% CIs, and diamonds represent pooled effect sizes [8,12,14,15,16,17,18].

The potential metabolic effects of sucralose consumption were further evaluated by pooling data on fasting blood glucose (FBG) and homeostatic model assessment for insulin resistance (HOMA-IR). The meta-analysis results for the metabolic outcomes are presented in Figure 4. Sucralose consumption was not associated with a statistically significant change in fasting blood glucose (FBG) (SMD = −0.04; 95% CI = 0.63 to 0.56; *p* = 0.88; I^2^ = 69.61%). Similarly, no significant effect was observed for HOMA-IR, a surrogate marker of insulin resistance (SMD = −0.13; 95% CI = −0.36 to 0.10; *p* = 0.21; I^2^ = 0.00%).

### 3.5. Summary of the Main Findings

The main findings of the quantitative and descriptive analyses are summarized in Table 3. Overall, the meta-analysis showed no statistically significant effects of sucralose consumption on alpha diversity, short-chain fatty acid (SCFA) concentrations, fasting blood glucose, or HOMA-IR. Descriptive findings for microbial compositions were inconsistent across studies.

## 4. Discussion

### 4.1. Effects of Sucralose Consumption on Gut Microbiota Composition

The findings of this systematic review demonstrated considerable heterogeneity among the included studies. The first source of heterogeneity was the variation in sucralose dosage and intervention duration across studies. In addition, the comparator formulations varied considerably among the ten included studies and may have contributed to variability in the reported outcomes. Based on their composition, the comparators were descriptively classified into two categories: Category A (sugar-free comparators) and Category B (sugar-containing comparators).

Category A included sugar-free comparators such as water, capsule-based formulations, and citric acid-containing solutions, whereas Category B included glucose-, maltodextrin-, or dextrose-containing formulations. Although sugar-free comparators may minimize the potential metabolic effects of nutritive carbohydrates, they should not necessarily be considered biologically inert. For example, citric acid and capsule excipients may exert biological effects, while sugar-containing comparators may independently influence glucose metabolism and gut microbial activity. Therefore, differences in comparator composition may represent an additional source of clinical heterogeneity. However, because the number of studies contributing to each outcome was limited, subgroup analysis according to comparator type was not performed.

The pooled analysis of alpha diversity, based on three studies, demonstrated no overall effect of sucralose consumption on gut microbial diversity (Hedges’ g = 0.00; 95% CI = −1.16 to 1.15; *p* = 0.992; I^2^ = 25.67%). The point estimate was essentially null, indicating no numerical difference in alpha diversity between the sucralose and control groups. This finding is consistent with Thomson et al. [8], who reported that seven days of sucralose consumption at 780 mg/day did not significantly alter gut microbial diversity. Similarly, Ruiz-Ojeda et al. [4] reported that the effects of non-nutritive sweeteners on alpha diversity were inconsistent and may depend on factors such as dose and duration of exposure.

However, the absence of a significant change in alpha diversity does not necessarily indicate that sucralose has no effect on specific bacterial taxa or overall microbial composition. Changes in the relative abundance of particular bacterial groups may occur without producing detectable changes in overall richness or evenness. Therefore, microbial composition should be considered separately from alpha diversity when interpreting the potential effects of sucralose.

Changes in microbial community structure were also reported through beta diversity. Several included studies, including Thomson et al. [8], Ahmad et al. [6], Mendez-Garcia et al. [12], Suez et al. [16], and Romo-Romo et al. [14], assessed beta diversity. However, differences in analytical approaches and the lack of comparable quantitative effect estimates prevented a formal meta-analysis. Therefore, beta diversity was interpreted descriptively rather than quantitatively. The available findings are consequently insufficient to establish whether sucralose consistently alters the overall structure of the gut microbial community.

Changes in the relative abundance of Firmicutes and Bacteroidetes were further summarized using the Firmicutes-to-Bacteroidetes (F/B) ratio. As shown in Table 3, three studies reported a numerical decrease in the F/B ratio following sucralose consumption, whereas Ahmad et al. [6] reported an increase. The increased F/B ratio reported by Ahmad et al. [6] was attributable to a greater abundance of Firmicutes compared with Bacteroidetes following the intervention. In contrast, Mendez-Garcia et al. [12] reported a significant change in Firmicutes (*p* = 0.03) without a significant change in Bacteroidetes, while Romo-Romo et al. [14] reported significant changes in both phyla. Thomson et al. [8] and Ahmad et al. [6] did not report statistically significant changes in either phylum.

These findings indicate that changes in the F/B ratio were inconsistent across studies and may reflect differential responses of individual bacterial groups rather than a uniform effect of sucralose on the gut microbiota. In addition, the F/B ratios were calculated from relative abundance data extracted from published figures and were not directly reported or statistically analyzed in the original studies. Therefore, these values should be interpreted descriptively rather than as evidence of statistically significant changes in the F/B ratio. The post hoc power analysis indicated low statistical power for the alpha diversity meta-analysis (5.0%), suggesting a limited ability to detect small effects (Table S5).

### 4.2. Effects of Sucralose Consumption on Short-Chain Fatty Acids

The present study also evaluated short-chain fatty acids (SCFAs), including acetate, propionate, and butyrate. SCFAs are important microbial metabolites produced through bacterial fermentation and play roles in intestinal barrier integrity, immune regulation, and host energy metabolism [20]. Although none of the SCFAs showed statistically significant pooled effects, the effect estimates demonstrated different numerical directions. Acetate showed a positive pooled effect (Hedges’ g = 1.45; 95% CI = −2.37 to 5.28; *p* = 0.130), indicating a numerical tendency toward higher acetate concentrations in the sucralose group than in the control group. Similarly, propionate showed a positive pooled effect (Hedges’ g = 0.66; 95% CI = −3.17 to 4.49; *p* = 0.272). However, the confidence intervals for both outcomes crossed zero. Therefore, these findings do not provide sufficient evidence that consumption of sucralose increases acetate or propionate concentrations.

In contrast, butyrate showed a negative pooled effect (Hedges’ g = −0.42; 95% CI = −2.46 to 1.62; *p* = 0.756), indicating a numerical tendency toward lower butyrate concentrations in the sucralose group compared with the control group. However, the confidence interval crossed zero, indicating that the pooled effect was not statistically significant. Furthermore, substantial heterogeneity was observed (I^2^ = 88.10%). Only two studies contributed to this analysis and reported opposite effect directions, with Ahmad et al. [6] reporting a positive estimate and Romo-Romo et al. [14] reporting a negative effect estimate. Thus, the pooled estimate of butyrate should be interpreted with caution due to a small number of studies, opposing effect directions, and high heterogeneity. The post hoc power analysis also indicated low statistical power for the SCFA outcomes, with estimated power of 5.0% for butyrate, 8.4% for acetate, and 6.3% for propionate (Table S5). These findings suggest that the available studies had limited ability to detect small effects.

The different numerical patterns observed among SCFAs may be related to differences in microbial composition and substrate availability. Li et al. [21] demonstrated in an in vitro fermentation model that differences in polysaccharide characteristics influenced microbial fermentation and SCFA production, accompanied by changes in bacterial composition. However, because this study investigated blackberry polysaccharides rather than sucralose and was conducted in vitro, its findings provide a mechanistic context rather than direct evidence of the effect of sucralose consumption in humans.

Overall, the present meta-analysis does not demonstrate a statistically significant effect of sucralose consumption on acetate, propionate, or butyrate concentrations. The observed directions should therefore be considered patterns in the available evidence rather than evidence of a consistent biological effect of sucralose on SCFA production.

### 4.3. Effects of Sucralose Consumption on Metabolic Outcomes

In addition to microbiota-related outcomes, the present study also evaluated metabolic responses by measuring fasting blood glucose (FBG) and the homeostatic model assessment of insulin resistance (HOMA-IR). Both pooled estimates were negative, although neither reached statistical significance. The pooled effect of FBG was negative (Hedges’ g = −0.04; 95% CI = −0.63 to 0.56; *p* = 0.882; I^2^ = 69.61%). Because the effect size was calculated as sucralose minus control, the negative point estimate indicates a very small numerical tendency toward lower FBG values in the sucralose group compared with the control group. However, the confidence interval crossed zero, and the effect was not statistically significant. Therefore, the result does not provide evidence of a significant effect of sucralose consumption on fasting blood glucose.

Similarly, HOMA-IR showed a negative pooled effect (Hedges’ g = −0.13; 95% CI = −0.36 to 0.10; *p* = 0.212; I^2^ = 0.00%), indicating a numerical tendency toward lower HOMA-IR values in the sucralose group than in the control group. Nevertheless, the confidence interval crossed zero, and the pooled effect was not statistically significant. Thus, the findings do not demonstrate a significant effect of sucralose consumption on insulin resistance as assessed by HOMA-IR. The post hoc power analysis showed low statistical power for both metabolic outcomes, with an estimated power of 5.2% for fasting blood glucose and 19.2% for HOMA-IR (Table S5). The relatively low power indicates that the available evidence may have had limited ability to detect small effects.

FBG and HOMA-IR showed negative pooled estimates, suggesting a slight tendency toward lower values in the sucralose group. However, the confidence intervals crossed zero, indicating that these differences were not statistically significant. Therefore, the findings do not support a clear effect of sucralose on blood glucose or insulin sensitivity.

The substantial heterogeneity observed for FBG may be related to differences in participant characteristics, sucralose dose, intervention duration, comparator formulation, and baseline metabolic status. These findings are consistent with previous clinical studies reporting no significant effects of sucralose on fasting glucose and other markers of glucose homeostasis [22,23]. Pepino et al. [24] reported that sucralose increased insulin secretion following an oral glucose tolerance test in individuals with obesity but did not significantly alter fasting blood glucose. In contrast, Suez et al. [5] reported that non-nutritive sweeteners could alter glucose tolerance through microbiota-mediated mechanisms during prolonged exposure. These findings suggest that intervention duration may be an important factor when evaluating the potential metabolic effects of non-nutritive sweeteners.

Recent experimental evidence also supports a potential relationship between gut microbiota, intestinal barrier function, and metabolic regulation. Chen et al. [25] demonstrated that modulation of gut microbiota and the intestinal mucosal barrier was associated with glucose-related outcomes in a mouse model of type 2 diabetes. Wu et al. [26] further reported associations between specific gut microbial taxa, microbial metabolites, and impaired glucose control. However, these studies involved different interventions and experimental models and therefore provide a mechanistic context rather than direct evidence of the metabolic effects of sucralose in healthy adults.

### 4.4. Potential Mechanisms Linking Gut Microbiota and Metabolic Responses

Based on the integrated data, several biological pathways may theoretically link changes in gut microbiota with metabolic responses following sucralose consumption. However, these mechanisms should be regarded as hypotheses rather than established mechanisms demonstrated by the present meta-analysis. The first potential pathway involves SCFA-mediated signaling. SCFAs may interact with G protein-coupled receptors such as GPR41 and GPR43, which are involved in enteroendocrine signaling and may influence the secretion of glucagon-like peptide-1 (GLP-1) and peptide YY (PYY). These pathways may subsequently influence glucose homeostasis and insulin sensitivity. However, because the present meta-analysis did not demonstrate statistically significant changes in acetate, propionate, or butyrate, this mechanism remains hypothetical.

A second potential pathway involves intestinal barrier function and inflammatory signaling. Alterations in gut microbial composition or microbial metabolites may influence intestinal barrier integrity and inflammatory responses. Chen et al. [25] demonstrated an association between gut microbiota, intestinal mucosal barrier function, and glucose metabolism in a diabetic mouse model. Li et al. [27] also reported that microbiome regulation was associated with intestinal barrier function and immune homeostasis in experimental models. Zhang et al. [28] further discussed interactions between gut microbiota, microbial metabolites, and intestinal inflammatory processes.

These findings provide biological plausibility for interactions between gut microbiota, microbial metabolites, intestinal barrier function, and host metabolism. Nevertheless, none of the randomized controlled trials included in the present review directly assessed GPR41/GPR43 signaling, intestinal permeability, LPS/TLR4 signaling, or related inflammatory pathways. Therefore, these mechanisms should be regarded as biologically plausible explanations rather than direct evidence of causality.

### 4.5. Study Limitations

Several factors may have influenced the interpretation of the meta-analysis findings. The studies used different sucralose doses, ranging from 48 to 780 mg/day, and intervention periods of one to ten weeks. Considerable variation was also found in the comparator formulations. We classified the comparators as sugar-free or sugar-containing for descriptive purposes, but the small number of studies did not allow us to examine differences between these comparator groups through subgroup analysis.

Only seven of the ten included studies had sufficient quantitative data to be included in the meta-analysis. The sample sizes were also generally small, which may have reduced the precision of the pooled estimates and contributed to the uncertainty of the findings. This was especially evident in the butyrate analysis, for which only two studies were available, and heterogeneity was high. Most of the interventions were also relatively short, with the majority lasting less than four weeks. As a result, these studies may not have captured changes in gut microbiota composition or metabolic responses that develop over a longer period. The findings, therefore, primarily reflect the short-term effects reported in the available studies and may not capture the effects of prolonged sucralose consumption.

Characteristics of participants also differed across studies, particularly in terms of age, sex distribution, baseline metabolic status, habitual dietary intake, and other lifestyle factors. Such differences may have affected gut microbiota composition and metabolic responses and may partly explain the variation observed between studies. The future of randomized controlled trials would benefit from more detailed reporting of participant characteristics, different sexes, and baseline metabolic status. Variation in the methods used to assess gut microbiota may have further contributed to differences between studies. The sequencing methods, sample collection procedures, and analytical pipelines were not uniform, which may influence the detection and interpretation of changes in the gut microbiota. Beta diversity was also excluded from the quantitative synthesis because comparable effect estimates were not consistently reported across studies.

The risk-of-bias assessment classified three studies as having a low overall risk of bias, four as having some concerns, and three as having a high risk of bias. The presence of some concerns and high risk of bias in several studies may have affected the reliability of the pooled estimates. Publication bias was not formally assessed because fewer than ten studies were available for each meta-analyzed outcome. With this small number of studies, funnel plot asymmetry and Egger’s regression test may provide unreliable results. Thus, publication bias could not be ruled out.

The statistical power of the quantitative synthesis was also limited. The post hoc power analysis indicated low power across the evaluated outcomes, ranging from 5.0% to 19.2%. This limitation may have reduced the ability of the meta-analysis to detect small effects and increased the possibility of a type II error. Therefore, the absence of statistically significant pooled effects should be interpreted with caution, particularly for outcomes supported by only a small number of studies.

Overall, the meta-analysis did not show significant changes in gut microbiota composition or metabolic outcomes following sucralose consumption in healthy adults. Some outcomes showed differences in the direction of the effect, but none of the pooled estimates were statistically significant. These results do not establish a significant effect of sucralose, but they also do not rule out biological effects. Further randomized controlled trials with longer intervention periods and more consistent sucralose doses and comparator formulations are needed. Future studies should also include a broader range of gut microbiota measures, microbial metabolites, and metabolic biomarkers, while considering factors such as sex and baseline metabolic status.

## 5. Conclusions

Based on 10 randomized controlled trials (RCTs) involving adult populations, with short-term sucralose interventions ranging from 1 to 10 weeks and doses of 48–780 mg/day, this systematic review and meta-analysis found no statistically significant changes in alpha diversity, fecal short-chain fatty acid (SCFA) concentrations, fasting blood glucose, or HOMA-IR following sucralose consumption. Although changes in gut microbiota composition, as assessed by the Firmicutes-to-Bacteroidetes (F/B) ratio, have been reported in several studies, these findings have been inconsistent, with only one study demonstrating a statistically significant change. A numerical tendency toward lower butyrate concentrations and fluctuations in the relative abundance of specific bacterial phyla were also observed in individual studies, although these findings were not sufficient to establish a consistent adverse effect. Overall, the available evidence from short-term RCTs does not indicate statistically significant changes in the microbiota and the evaluated metabolic outcomes. However, these findings are insufficient to establish the long-term safety of sucralose consumption. Further well-designed RCTs with longer intervention periods, more standardized comparator formulations, and comprehensive assessment of inflammatory markers and intestinal permeability are warranted to better characterize the potential long-term effects of sucralose on gut microbiota and metabolic health.

## Figures and Tables

**Table 1:** Characteristics of sucralose consumption studies included in the systematic review.

Country	n = C/I	Doses of Sucralose (mg/day)	Duration (Weeks)	Type of Comparator	Parameter	Key Findings	Study
Mexico	20/20	48	10	A: Unadulterated water	Plasma glucose, insulin, beta diversity	No changes in glucose tolerance or insulin.	Mendez-Garcia et al. [12]
Mexico	34/61	48–96	5	A: Non-nutritive sweetener drinks	Gastrointestinal syndromes	No significant treatment-related effects observed.	Mendoza-Martinez et al. [13]
Mexico	10/10	270	4	A: Cornstarch capsule	Plasma glucose, HOMA-IR, alpha diversity, beta diversity, short-chain fatty acids (SCFA)	No significant changes in glucose metabolism.	Romo-Romo et al. [14]
Thailand	15/15	200	4	A: Empty gelatin capsule	Plasma glucose, HOMA-IR	No significant changes in insulin sensitivity.	Lertrit et al. [15]
Israel	23/21	102	3	B: Glucose sachet	Plasma glucose, insulin, beta diversity	Individual glycemic responses were altered.	Suez et al. [16]
USA	10/11	48	3	B: Glucose solution	Plasma glucose, insulin sensitivity	No significant changes in glucose or insulin levels.	Nichol et al. [17]
Canada	8/8	136	2	A: A mixture of water, citric acid, and lemon	Alpha diversity, beta diversity, short-chain fatty acids (SCFA)	No significant effects on FBG or HOMA-IR	Ahmad et al. [6]
Mexico	30/30	120–157	2	B: Dextrose and maltodekstrin sachet	Plasma glucose, HOMA-IR	Fasting glucose and insulin remained unchanged.	Romo-Romo et al. [18]
Chile	17/17	780	1	A: Capsule containing 250 mg of CaCO_3_	HOMA-IR, beta diversity	No significant changes in glucose metabolism.	Thomson et al. [8]
USA	8/10	204	1	A: Unsweetened carbonated water	Sucralose concentration in urine	Urinary sucralose confirmed participant compliance.	Sylvetsky et al. [19]

Abbreviations: C = control; I = intervention. Comparator categories: A = sugar-free comparator; B = sugar-containing comparator.

**Table 2:** Changes in the Firmicutes-to-Bacteroidetes (F/B) ratio reported in the included studies.

F/B Before	F/B After	Fold Changes	Significance	Study
5.18	4.41	0.85	F: *p* > 0.05 B: *p* > 0.05	Thomson et al. [8]
0.50	2.32	4.65	F: *p* = 0.18 B: *p* = 0.61	Ahmad et al. [6]
1.00	0.46	0.46	F: *p* = 0.03 B: *p* > 0.05	Mendez-Garcia et al. [12]
1.42	0.97	0.68	F: *p* < 0.05 B: *p* < 0.05	Romo-Romo et al. [14]

Abbreviations: F = *Firmicutes*; B = *Bacteroidetes*. The F/B ratio was estimated from relative abundance data extracted by graph digitization using WebPlotDigitizer. As these values were not explicitly reported or statistically analyzed in the original studies, the calculated F/B ratios were used exclusively for descriptive purposes to illustrate the direction of changes in gut microbiota composition rather than for statistical inference.

**Table 3:** Summary of the main findings of the meta-analysis.

Outcome	Synthesis	N of Studies	*Hedges’ g*	95% CI	*p*-Value	I^2^ (%)	Main Finding
Alpha diversity	Meta-analysis	3	−0.00	−1.16 to 1.15	0.99	25.67	No significant effect
F/B ratio	Descriptive	4	-	-	-	-	Inconsistent direction across studies
Acetate	Meta-analysis	2	1.45	−2.37 to 5.28	0.13	0.00	No significant effect
Propionate	Meta-analysis	2	0.66	−3.17 to 4.49	0.27	31.49	No significant effect
Butyrate	Meta-analysis	2	−0.42	−2.46 to 1.62	0.76	88.10	No significant effect; high heterogeneity
Fasting blood glucose	Meta-analysis	7	−0.04	−0.63 to 0.56	0.88	69.61	No significant effect
HOMA-IR	Meta-analysis	5	−0.13	−0.36 to 0.10	0.21	0.00	No significant effect

Abbreviations: F/B, Firmicutes-to-Bacteroidetes ratio; CI, confidence interval; I^2^, heterogeneity statistic; HOMA-IR, homeostatic model assessment of insulin resistance. Negative effect estimates indicate lower outcome values in the sucralose group compared with the control group because effect sizes were calculated as sucralose minus control.

## Data Availability

The contributions presented in this study are included in the article. Further inquiries can be directed to the corresponding author, Nuri Andarwulan.

## Supplementary Materials

The following supporting information can be downloaded at https://www.mdpi.com/article/10.3390/ijms27188377/s1.

## Abbreviations

The following abbreviations are used in this manuscript:

ADI	Acceptable Daily Intake
BMI	Body Mass Index
CAGR	Compound Annual Growth Rate
EFSA	European Food Safety Authority
F/B	*Firmicutes*-to-*Bacteroidetes*
FDA	Food and Drug Administration
GLP-1	Glucagon-like peptide-1
GPR	G protein-coupled receptor
HbA1c	Glycated hemoglobin
HOMA-IR	Homeostatic Model Assessment for Insulin Resistance
LPS	Lipopolysaccharide
OGTT	Oral glucose tolerance test
PYY	Peptide YY
RCT	Randomized controlled trial
SCFA	Short-chain fatty acid
SMD	Standardized Mean Difference
